# League of Mercy

**Published:** 1904-03-19

**Authors:** 


### LEAGUE OF MERCY.

Harrod's Stores has succeeded rapidly because of the enterprise of its management. On Tuesday night a new departure was made by the Managing Director, Mr. R. Burbidge, who invited the employés, some 2,500 in number, to hear a speech by Sir Henry Burdett on the London Hospitals, in the Great Hall under the dome, which was crowded by an attentive and deeply interested audience. Lord Kilmorey presided, and Dora Lady Chesterfield, President of the South Kensington Division of the League of Mercy, was also present, and spoke. In the result it was settled that Harrod's Stores should lead the way among great houses of business by forming a branch of the League of Mercy, it being hoped that each superintendent of a department, of which there are 60, will become a vice-president and secure at least 20 members from the employés, who will render in the days of health regular service in the cause of the sick. The meeting closed the winter series of speeches Sir Henry Burdett has been making throughout London, and the meeting was probably the most successful one ever held.

